# Profiling the Bioactive Compounds in Broccoli Heads with Varying Organ Sizes and Growing Seasons

**DOI:** 10.3390/plants13101329

**Published:** 2024-05-11

**Authors:** Lu Shi, Yahui Li, Menghua Lin, Ying Liang, Zhiyong Zhang

**Affiliations:** 1 Jiangsu Key Laboratory for Food Quality and Safety-State Key Laboratory Cultivation Base, Ministry of Science and Technology, Jiangsu Academy of Agricultural Science, Nanjing 210014, China; lus202010@163.com (L.S.); liqianhao217@126.com (Y.L.); 20220017@jaas.ac.cn (M.L.); 2 School of Food and Biological Engineering, Jiangsu University, Zhenjiang 212000, China

**Keywords:** *Brassica oleracea*, phenolic compounds, glucoraphanin, minerals, phytosterols

## Abstract

Broccoli is a rich source of diverse bioactive compounds, but how their contents are influenced by different growing seasons and variations in broccoli head sizes remains elusive. To address this question, we quantified sixteen known bioactive compounds and seven minerals in broccoli with varying head sizes obtained in two different growing seasons. Our results suggest that the contents of vitamin C, total phenols, carotenoids, and glucoraphanin were significantly higher in samples from the summer–autumn season, showing increases of 157.46%, 34.74%, 51.80%, and 17.78%, respectively, compared with those from the winter–spring season. Moreover, chlorogenic acid is a phenolic compound with relatively high contents among the six detected, while beta-sitosterol is the sterol with relatively high contents. Further, principal component analysis was conducted to rank the comprehensive scores of the profiles of phenolic compounds, phytosterols, and minerals, demonstrating that the broccoli samples grown during the summer–autumn season achieved the highest composite scores. Our results indicate that broccoli heads from the summer–autumn season are richer in a combination of bioactive compounds and minerals than those from the winter–spring season based on the composite score. This study extends our understanding of the nutrition profiles in broccoli and also lays the foundation for breeding broccoli varieties with improved nutrition quality.

## 1. Introduction

Vegetables are essential components of a balanced human diet. Growing evidence indicates that the daily consumption of vegetables plays an important role in the prevention of many chronic diseases through a wide range of mechanisms [1,2]. Broccoli (*Brassica oleracea* var. *italica*) is one of the most commonly consumed vegetables worldwide, as manifested by the record that over 25.84 million tons of broccoli and cauliflowers were produced globally in the year 2021 (FAOSTAT, 2023).

Broccoli belongs to the Brassicaceae family, the members of which provide a significant source of bioactive components, such as vitamins, glucosinolates, polyphenols, and minerals [3,4,5]. Glucosinolates are anionic sulfur-rich secondary metabolites found in vegetables belonging to the brassicaceae and related families [6]. Glucosinolates and their hydrolyzed products have been recognized as having antimicrobial, anticancer, and anti-inflammatory bioactivities [7,8]. Various antioxidants including vitamins, polyphenols, and carotenoids are also abundant in broccoli. In particular, vitamin C is a water-soluble and powerful antioxidant, whose nutrients are needed for the body to form blood vessels, cartilage, muscle, and collagen in the bones [9]. Polyphenols are a series of metabolites biosynthesized via the phenylalanine metabolic pathway [10], playing important roles in nutrient acquisition, enzyme activity, and photosynthesis in plants [11,12]. Carotenoids are natural tetraterpenoid pigments with an essential role in photosynthesis [13]. Phytosterols, as another class of natural health-beneficial products, have strong anti-inflammatory properties and have been associated with reduced risks of cardiovascular and heart attack and maintaining blood cholesterol levels [14,15]. Excessive blood cholesterol is associated with an increased risk of cardiovascular diseases [16]. This is because high levels of low-density lipoprotein cholesterol in the blood can lead to arterial hardening, thereby increasing the risk of heart disease and stroke. However, this association is mainly related to the intake of cholesterol from animal foods rather than plant-based foods [17]. Plants contain cholesterol, although the concentration of cholesterol is relatively low [18].

Broccolis are usually grown in two seasons of the year in southern China: in late winter to early spring, or in late summer to early fall. The two growing seasons are different in temperature and light, which affect the accumulation of bioactive compounds in broccoli [19]. Seasonal variations in the accumulation of some bioactive compounds have been reported in vegetables [20]. Commercial cabbages purchased in early fall have higher levels of isothiocyanates compared with those purchased late in the year [20]. Thus, it is necessary to make a comprehensive assessment of the effects of the two growing seasons on the accumulation of various bioactive compounds in broccoli.

Most previous studies on bioactive components in broccoli have focused on how to maintain them after harvest or how to minimize losses during broccoli processing [21,22]. Nonetheless, the correlation between the growing and harvesting time on the content of these bioactive compounds in broccoli heads remains unclear. In this research, we aim to comprehensively analyze the effects of growing season and diameter size on the nutritional qualities of broccoli heads. Specifically, the contents of various bioactive compounds were examined by biochemical experiments in broccoli heads from two different growing seasons and with different sizes. Our comprehensive assessment of the contents of various bioactive compounds in broccoli heads expands our understanding of the accumulation of these bioactive compounds in broccoli heads and provides guidance for broccoli production.

## 2. Results and Discussion

### 2.1. Soluble Soilds and Dry Matter Contents in Broccoli Heads Are Influenced by Growing Seasons

Total soluble solids (TSS) and dry matter contents are key indicators of quality widely used for various vegetables and fruits [23,24]. We measured the TSS and dry matter contents of broccoli from two growing seasons in broccoli head samples with different head sizes (Table 1). TSS and dry matter contents of broccoli heads of the winter–spring growing season were significantly higher than those of the summer–autumn growing season. These results are consistent with the reported influence of different cultivation times on dry matter and total sugar contents in cucumber [25]. We reason that the lower TSS in broccoli grown in the summer–autumn growing season may be a consequence of the relatively higher temperatures because high temperatures during the growing stages often decrease the accumulation of TSS [26,27]. During the winter–spring growing season, environmental conditions are conducive to the accumulation of higher levels of TSS and dry matter in broccoli. Lower temperatures reduce the rate at which photosynthates are consumed through respiration, promoting the accumulation of sugars. Moreover, abundant light intensity during these seasons enhances photosynthetic efficiency, ensuring efficient synthesis of TSS. The TSS detected in small broccoli heads was significantly higher than in large broccoli heads. There was no significant difference in dry matter contents (Table 1).

### 2.2. Vitamin C, Total Phenols, Carotenoids, and Individual Phenolic Compound Contents in Broccoli Heads Are Influenced by Growing Seasons and Organ Size

**Figure 1:**
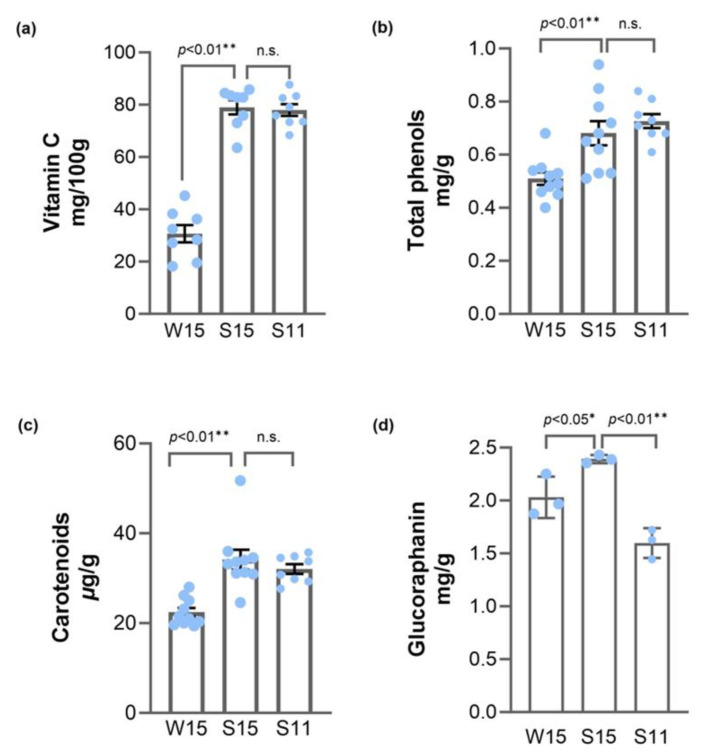
The comparison of vitamin C (**a**), total phenolic (**b**), carotenoid (**c**), and glucoraphanin (**d**) content in broccoli head samples with different growing seasons and organ sizes. W15, the samples grown in the winter–spring growing season with a 15 cm diameter head size. S15, the samples grown in the summer–autumn growing season with a 15 cm diameter head size. S11, the samples grown in the summer–autumn growing season with an 11 cm diameter head size. Student’s *t*-test is used to compare the data of W15 and S15, S15, and S11; dots, individual data points. Significant difference at ** *p* < 0.01 or * *p* < 0.05, n.s., no significant difference.

Vitamin C, phenolics, and carotenoids process antioxidant activity and thus play a positive role in maintaining human health [28,29,30,31]. As shown in Figure 1, the average contents of vitamin C, total phenols, and total carotenoids are 62.55 mg/100 g, 64.74 mg/100 g, and 30.42 mg/100 g. The contents of vitamin C, total phenols, and total carotenoids exhibited a seasonal-dependent trend (Figure 1), with significantly higher content in those grown in the summer than those grown in the winter. However, there was little difference in the accumulation of vitamin C, polyphenols, and carotenoids in broccoli heads of the two size groups.

The levels of vitamin C, total phenols, and carotenoids are influenced by the growing seasons, with the summer–autumn growing season more favorable for broccoli production. Several studies have reported that bioactive compounds in plants are influenced by various environmental factors [22,32]. The synthesis of these antioxidants is closely related to external temperature and light [33,34,35]. Our results show that broccoli harvested in October accumulated more vitamin C, polyphenols, and carotenoids, which may be attributed to the sufficient light and the favorable environment for the accumulation of these substances. In conclusion, the three antioxidants measured in broccoli heads were influenced by growing seasons, and the broccoli with a growth cycle of summer–autumn accumulated more of these antioxidants compared to the winter–spring growing season.

Regarding the individual phenolic compound, six phenolic compounds, including ferulic acid, catechin, quercetin, caffeic acid, chlorogenic acid, and rutin, in broccoli heads were quantified (Table 2). The concentration of chlorogenic acid was found to be the most abundant of the six measured phenolics, with an average value ranging from 128.70 μg/g to 162.41 μg/g, and the head size had a significant impact on the variation of chlorogenic acid content. The results showed that the chlorogenic acid content in broccoli with a diameter of 15 cm was significantly higher than that in broccoli with a diameter of 11 cm (Table 2). 

The seasonal variation in the concentration of phenolic compounds, such as ferulic acid, catechin, and quercetin in broccoli heads, with higher levels observed in the summer–autumn growing seasons compared to winter–spring, indicates a significant influence of environmental factors on the plant’s secondary metabolism [36]. The high-temperature and high-humidity environment of the summer–autumn growing seasons increase plant diseases [37]. Phenolic compounds, like ferulic acid and quercetin, are important components involved in the phenylpropanoid metabolic pathway [38,39]. The accumulation of phenolic compounds can scavenge reactive oxygen species within the plants and inhibit lipid peroxidation, thereby enhancing the plant’s antioxidant capacity and disease resistance, further protecting the plants. Therefore, the higher levels of total phenolic content, as well as the other five types of phenolic monomers observed in broccoli heads grown in the summer–autumn seasons, may be related to that plants produce a large number of phenolic compounds to strengthen cell wall structures and resist pathogen invasion when they respond to biotic stress [40].

To further investigate the effect of broccoli head size on the content of six phenolic compounds during the summer–autumn growing seasons, we compared the levels of these compounds in broccoli heads of different sizes. Notably, the concentration of chlorogenic acid was significantly higher in 15 cm broccoli head samples compared to 11 cm broccoli head samples (Table 2). In contrast, the content of ferulic and rutin exhibited the opposite trend across varying head sizes. When considering the total phenolic content, our findings suggest that the size of the broccoli head may influence the levels of specific phenolic compounds rather than total phenolic content.

Together, PCA was conducted to rank the comprehensive scores of the profiles of phenolic compounds, demonstrating that the broccoli samples grown during the summer–autumn season with a diameter of 11 cm achieved the highest composite scores (Table S4).

### 2.3. Glucoraphanin Content in Broccoli Heads Is Influenced by Growing Seasons and Organ Size

Glucoraphanin, an abundant glucosinolate of broccoli samples [28], was identified by HPLC-DAD. As shown in Figure 1d, the concentration of glucoraphanin in broccoli heads ranged from 1.44 mg/g to 2.43 mg/g. Broccoli head samples from the summer–autumn growing season (S15) were significantly higher than those grown in winter–spring (W15) (Figure 1d). These results reflect that changes in the growing season affect the accumulation of glucoraphanin in broccoli heads, which is consistent with those observed by [41]. Similarly, glucosinolate contents also exhibit significant seasonal variation in other crops, such as radish [42], baby leaf rapeseed [43], and cabbage [20]. We also found that the content of glucoraphanin is significantly higher in broccoli heads with a 15 cm diameter (S15) compared to broccoli heads with an 11 cm diameter (S11) (Figure 1d). 

Previous studies have documented that pre-harvest growth temperature has a strong influence on glucosinolate biosynthesis [44,45]. For example, it has a higher glucosinolate content in young cabbages grown under 30 °C compared with under 20 °C [46]. Therefore, our observation of the higher amount of glucoraphanin in the growing season of summer–autumn samples can be due to the higher temperature during growth (6~32 °C) compared with those grown in the relatively low temperatures (3~24 °C) of the winter–spring growing season. Specifically, glucosinolates are a group of sulfur-containing secondary metabolites that play an important role in plant defense against pests and pathogenic infections [47]. The higher temperature can potentially increase the incidence of plant diseases [37]. When plant tissues are damaged, glucosinolates are hydrolyzed by the enzyme myrosinase to produce a range of bioactive breakdown products, such as isothiocyanates, thiocyanates, and nitriles [47]. These compounds contribute to impeding the penetration of pathogens [47,48].

### 2.4. Phytosterol Profiles in Broccoli Heads Are Influenced by Growing Seasons and Organ Size

**Figure 2:**
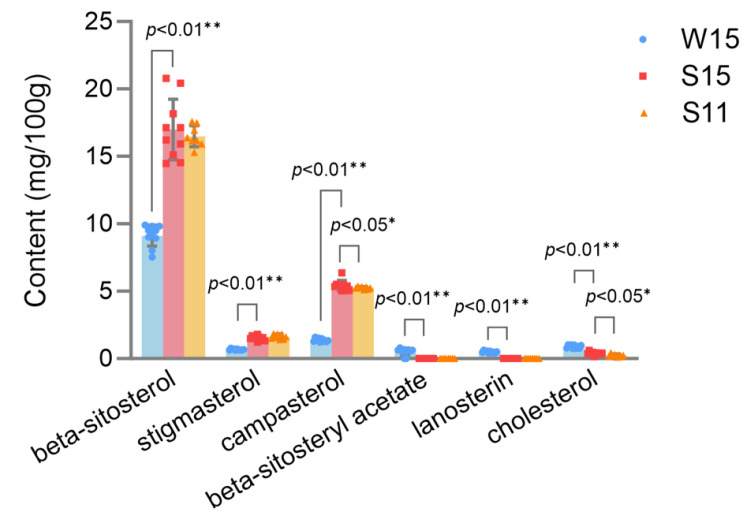
A comparison of six kinds of phytosterols content in broccoli head samples with different growing seasons and organ sizes. W15, the samples grown in the winter–spring growing season with a 15 cm diameter head size. S15, the samples grown in the summer–autumn growing season with a 15 cm diameter head size. S11, the samples grown in the summer–autumn growing season with an 11 cm diameter head size. Student’s *t-*test is used to compare the data of W15 and S15, S15 and S11; dots, individual data points. Significant difference at ** *p* < 0.01 or * *p* < 0.05.

Individual phytosterol compounds identified in two organ sizes of broccoli heads and different growing seasons are shown in Figure 2. A total of six phytosterols were detected in broccoli heads, namely, beta-sitosterol, stigmasterol, campesterol, beta-sitosteryl acetate, lanosterin, and cholesterol. In general, beta-sitosterol was the most dominant phytosterol among the broccoli heads, ranging from 5.67 to 20.78 mg/100 g, followed by compasterol (0.7 to 6.37 mg/100 g) and stigmasterol (0.48 to 2.38 mg/100 g) (Figure 2). Among these phytosterols, beta-sitosterol acetate, lanosterin, and cholesterol were low or nearly undetectable in broccoli heads. The phytosterol profiles identified in the present study are consistent with previous reports that beta-sitosterol is the predominant phytosterol in mustard leaves, accounting for over 80% of the total phytosterols [15].

Higher contents of beta-sitosterol, stigmasterol, and campesterol were characterized as grown in the summer–autumn growing season compared to those grown in the winter–spring growing season (Figure 2). Cholesterol is significantly higher in broccoli heads with a 15 cm diameter compared to broccoli heads with an 11 cm diameter (Figure 2). Seasonal variation exhibited a great effect on the accumulation of phytosterols. Similarly, the year-to-year variation in the content of phytosterol was reported in rye [49], which was possibly due to the impact of environmental factors, such as weather, soil, and precipitation. Based on meteorological data, monthly rainfall in October in Xiangshui County in 2021 was about three times higher than that in April. Therefore, rainfall may enhance phytosterol accumulation in broccoli heads, which is consistent with the results of [49]. They found that low rainfall during the month prior to the harvest caused a decrease in phytosterol accumulation in kernels [49]. Further, PCA was conducted to rank the comprehensive scores of the phytosterol profiles, demonstrating that broccoli samples cultivated during the summer–autumn season with a diameter of 11 cm attained the highest composite scores (Table S6). 

Collectively, we established the HPLC method and identified four kinds of phytosterols that were relatively more abundant in broccoli heads, while most of the previous studies on the composition and contents of phytosterols have been explored in oilseeds, nuts, and grains [50]. Although the content of phytosterols in broccoli heads is one to two orders of magnitude lower than those for oilseeds or cereal grains [50], the phytosterols in broccoli heads increase their nutritional diversity. This study of phytosterols in broccoli heads would provide data support for the better utilization of the bioactive components.

### 2.5. Mineral Contents in Broccoli Heads Are Influenced by Growing Seasons and Organ Size

Minerals are vital components in our foods and have multiple functions in keeping the body’s homeostasis and maintaining an active immune system [51]. Seven kinds of minerals were measured in broccoli heads from two growing seasons with two different head sizes (Table S4). Phosphorus, potassium, magnesium, calcium, and iron account for most of the minerals in broccoli heads, which ties well with the daily mineral requirements for an adult [52].

**Figure 3:**
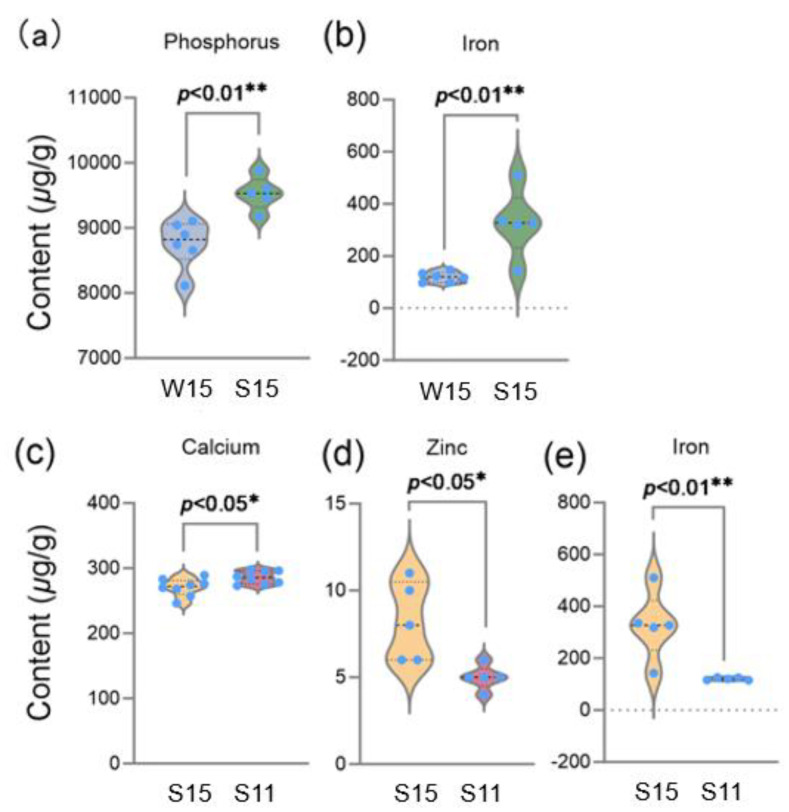
A comparison of the mineral content in broccoli head samples with different growing seasons and organ sizes. (**a**), a comparison of the content of phosphorus in W15 and S15 samples. (**b**), a comparison of the content of iron in W15 and S15 samples. (**c**), a comparison of the content of calcium in S15 and S11 samples. (**d**), a comparison of the content of zinc in S15 and S11 samples. (**e**), a comparison of the content of iron in S15 and S11 samples. W15, the samples grown in the winter–spring growing season with a 15 cm diameter head size. S15, the samples grown in the summer–autumn growing season with a 15 cm diameter head size. S11, the samples grown in the summer–autumn growing season with an 11 cm diameter head size. Student’s *t*-test is used to compare the data of W15 and S15, S15 and S11; dots, individual data points. Significant difference at ** *p* < 0.01 or * *p* < 0.05.

The amount of phosphorus, magnesium, and iron is higher in broccoli heads from those grown in the summer–autumn growing season than those grown in the winter–spring growing season (Table S4). Moreover, the levels of phosphorus and iron showed significant differences in broccoli heads from the two growing seasons (Figure 3a,b), indicating that the summer–autumn growing season is preferred for the accumulation of these elements in broccoli heads, which is in line with a previous report in cucumber fruits in which it was found that different cultivation seasons contribute to mineral element accumulation [25]. Zinc and iron contents exhibited a significant difference between the 15 cm and 11 cm diameter of the broccoli heads (Figure 3d,e). The amount of calcium is significantly higher in broccoli heads with an 11 cm diameter compared to broccoli heads with a 15 cm diameter (Figure 3c). The zinc, iron, and calcium contents of broccoli heads did not significantly differ between the winter–spring and summer–autumn growing seasons.

Broccoli has excellent nutritional potential, containing a variety of essential minerals that contribute to overall health. While it is widely recognized that the bioavailability of minerals from plant sources, such as iron, is typically lower compared to animal sources due to differences in mineral forms and the presence of antinutritional factors, research indicates that broccoli contains non-heme iron with relatively higher bioavailability among plant foods [53]. In addition, phosphate is also essential for plant growth and fitness [54]. During the summer–autumn growing season, higher temperatures may increase microbial activity, which accelerates the decomposition of organic matter, leading to greater phosphorus availability in the soil.

Taken together, broccoli is a valuable vegetable for a balanced diet. For the PCA comprehensive scores of seven minerals, it was found that broccoli samples from the summer–autumn season with a diameter of 15 cm had the highest composite scores (Table S7). A comparison of various minerals in broccoli heads from the two growing seasons with two different head sizes may provide data for broccoli production.

## 3. Materials and Methods

### 3.1. Plant Materials

Broccoli cultivar (Hanxiu) was grown in two seasons, late winter and late summer, and harvested in two seasons, spring (27 April 2021) and autumn (20 October 2021), separately. The two growing cycles were marked winter–spring and summer–autumn. These samples were planted in Xiangshui County (119°29′ E, 33°56′ N, Jiangsu, China). The temperature information can be found in Table S1. Then, the samples were transported to the Jiangsu Academy of Agricultural Sciences (118°51′ E, 32°2′ N, Nanjing, China) under cooled conditions. Samples collected from individual sites served as biological replicates. Three plants were collected from each site. Samples harvested in the autumn were divided into two groups based on the size of the diameter of the broccoli heads: 11 ± 1 cm (marked S11) and 15 ± 1 cm (marked S15). Then, the samples were cut into small pieces. One portion was ground in liquid nitrogen and then kept at −80 °C in a freezer for further analysis, while the others were immediately homogenized using a Conair Waring Laboratory Blender for 1 min to determine the total soluble solids and dry matter contents. 

The different head size experiments were only carried out for broccoli harvested during the summer–autumn growing seasons (marked S11 and S15). The different growing season experiments were carried out as follows: broccoli samples from the winter–spring season with a diameter of 15 cm (marked W15) and broccoli samples from the summer–autumn season with a diameter of 15 cm (marked S15).

### 3.2. Sample Preparation and Determinations

#### 3.2.1. Determination of Total Soluble Solids and Dry Matter Contents

The homogenized broccoli samples were filtered through double-layer gauze fabric. The level of total soluble solids in the filtrate was measured by a portable refractometer following the instructions. The results were recorded as a percentage (%). Dry matter contents were determined using 100 g of homogenized broccoli heads and dried in an oven until a constant weight at 60–70 °C. 

#### 3.2.2. Analysis of Vitamin C, Total Polyphenol, and Carotenoids

The vitamin C content was measured by the 2,6-dichloroindophenol titration method [55]. 

Total polyphenol contents were determined using the Folin–Ciocalteu method [56,57]. Briefly, samples (~1 g) were dissolved in 5 mL of 70% methanol. After vortexing, the samples were ultrasonicated for 30 min and then centrifuged at 8000 g for 5 min. Then, 0.5 mL of supernatant was moved to a 10 mL volumetric flask with 3 mL of distilled water and 500 μL Folin–Ciocalteu (0.5 N). After vortexing for 3 min, 1 mL of sodium carbonate was added to stop the reaction. The mixture was shaken and incubated for 2 h in a water bath at 30 °C. The absorbance was measured at 760 nm. The obtained calibration curve had an *R*^2^ value of 0.9907.

The carotenoid contents were evaluated as described previously with minor modifications [58]. In brief, samples (~1 g) were diluted to 10 mL of ethanol (0.01% butylated hydroxytoluene, BHT). Then, the mixture was ultrasonicated for 20 min to facilitate carotenoid extraction. After the extraction period, the samples were passed through a microporous membrane (pore size, 0.22 μm). The collected filtration measured the absorbance at 450 nm using a spectrophotometer. The carotenoid contents were calculated using Equation (1), according to the standard curve of bate-carotene.

(1) *C* = (*A*450 − 0.0297)/79.057 × 10/*W*

where C represents the carotenoid contents. *A*450 refers to the absorption readings at 450 nm, and W represents the weight of the samples.

#### 3.2.3. Analysis of Individual Phenolic and Glucosinolate Compounds by HPLC with Diode Array Detection (DAD)

For the chromatographic determination of individual phenolic compounds, phenolic compounds were extracted according to [59]. Glucosinolates were extracted as described by [60] with modifications. Briefly, 0.5 g of frozen broccoli powder was extracted with 1.5 mL of 70% (*v*/*v*) methanol in an ultrasonic bath for 10 min, and then samples were transferred to a water bath (70 °C) for 30 min, shaking every 5 min using a vortex stirrer. After centrifuging (12,000× *g*, 15 min, 4 °C), the supernatants were collected and dried using a nitrogen blower. The dried samples were re-dissolved in 1 mL of ultrapure water and filtered through a 0.22 μm polyvinylidene fluoride (PVDF) membrane. Both the phenolic acid and glucisinolate extracts were analyzed by HPLC-DAD in an Agilent 1260 Infinity Series system (Agilent Technologies, Wilmington, DE, USA). Chromatographic separations were achieved on a Polaris reverse-phased C18 column (250 × 4.6 mm, 5 μm). The injection volume was 10 μL. 

Chromatograms were recorded at 280 nm for phenolic compounds. The mobile phases consisted of 1% formic acid water (phase A) and methanol (phase B). The following mobile phase gradient was used at a constant flow rate of 0.8 mL/min: 5–10% B for 5 min; 10–20% B for 10 min; 20–30% B for 10 min; 30% B for 10 min; 30–50% B for 10 min; 50–60% B for 10 min; and 60–5% B for 5 min [61]. Ferulic, catechin, quercetin, caffeic acid, chlorogenic acid, and rutin were used as the standards. The individual phenolic compound content is expressed as μg/g on a fresh weight (FW) tissue based on the standards of phenolics.

Glucosinolates were separated during a 40 min chromatographic run. The mobile phases consisted of Milli-Q water (phase A) and acetonitrile/0.08 M sodium dihydrogen phosphate (12/88, *v*/*v*) (phase B). The mobile phase gradient used has been described by [62]. Glucosinolates were monitored at 227 nm. For the quantitative analysis of glucosinolates, glucoraphanin was used as the standard for glucosinolates. The glucoraphanin content is expressed as mg/g on a fresh weight (FW) tissue based on the standards of glucoraphanin. The standard curves of ferulic, catechin, quercetin, caffeic acid, chlorogenic acid, rutin, and glucoraphanin are shown in Table S2.

#### 3.2.4. Analysis of Individual Phytosterol Compound by HPLC-MS

Ground broccoli head samples were placed into an Eppendorf tube (50 mL) containing 30 mL of ethanol. After vortexing and shaking for 3 min, the samples were ultrasonicated for 60 min using an ultrasonic cleaner (Xianou Instrument Co., Nanjing, China) and then centrifuged at 8000× *g* for 10 min when the samples cooled down. The supernatant passed through a 0.22 μm PVDF membrane.

The phytosterol was analyzed by an Agilent 1260 Series HPLC system (Agilent Technologies, Wilmington, DE, USA). The analytes were separated by an Agilent ZORBAX RRHD Eclipse Plus C18 column (50 × 3 mm, 1.8 μm) held at 30 °C. The mobile phase ratio of methanol/water (90:10% *v*/*v*) maintains a flow rate of 0.5 mL/min with a temperature of 350 °C. The injection volume was 10 μL. The identification and quantification parameters of mass spectrometry are shown in Table S2. The mass spectrum detector was operated in positive electrospray ionization (ESI).

#### 3.2.5. Measurement of Mineral Content

The mineral assay was carried out referring to the method reported by [63], with some modifications. Briefly, 0.4 g of sample was digested in concentrated sulfuric acid for 30 min in a microwave digestion system (350 °C) for element extraction. Next, add hydrogen peroxide 2 to 3 times (5–10 drops per time) during the digesting stage. Phosphorus concentration was determined using an automated chemistry analyzer system (SmartChem 200, Kopermann Analytical Instruments Co., Beijing, China). Potassium, zinc, sodium, magnesium, calcium, and iron concentrations were detected by an atomic absorption spectrometer (PinAAcle 900T, PerkinElmer Inc., Waltham, MA, USA). 

#### 3.2.6. Statistical Analysis

All experiments were carried out at least three times independently. Statistical analyses were performed using Student’s *t*-test. The results were expressed as mean ± standard error of the mean (SEM). The principal component analysis (PCA) and calculation of comprehensive scores according to the main factors were carried out using IBM SPSS statistics software. The *t*-test analysis and graphs generated were conducted using GraphPad Prism 10.1.0 software.

## 4. Conclusions

We utilized biochemical methods to measure sixteen bioactive substances and seven mineral elements with different growing seasons and head sizes of broccoli heads. Our results suggest that the contents of vitamin C, total phenols, carotenoids, and glucoraphanin were significantly higher in samples from the summer–autumn season compared with those from the winter–spring season. In addition, chlorogenic acid, rutin, and catechin are the phenolic compounds with relatively high contents among the six detected, while beta-sitosterol, campesterol, and stigmasterol are the sterols with relatively high contents.

Our results indicate that broccoli heads from the summer–autumn growing season are richer in a combination of bioactive compounds and mineral elements than those from the winter–spring season based on their composite scores. However, the various bioactive compounds and mineral elements in broccoli head samples are not consistently related to the size of the broccoli head. Compared to the winter–spring season, the summer–autumn season experiences higher temperatures, which can lead to an increase in pests and diseases. Broccoli may accumulate a higher number of bioactive compounds and mineral elements as a response to environmental changes. In the future, the impact of various environmental changes on the bioactive substances in broccoli could be further investigated. This study extends our understanding of the nutrition profiles in broccoli, providing guidance for broccoli production. By elucidating the ideal growing season and head size, this study also lays the foundation for breeding broccoli varieties with improved nutrition quality.

## Figures and Tables

**Table 1:** Total soluble solids (TSS) and dry matter contents of broccoli head samples from different growth seasons and head sizes.

Samples	TSS (%)	Dry Matter Contents (%)
W15	7.50 ± 0.47	11.88 ± 0.75
S15	6.13 ± 0.33	11.13 ± 0.65
S11	6.44± 0.26	11.56 ± 0.68
W15 vs. S15	*p* < 0.01	*p* < 0.05
S15 vs. S11	*p* < 0.05	n.s.

Notes: The data are presented as mean ± standard error of the mean (SEM). W15, the samples grown in the winter–spring growing season with a 15 cm diameter head size. S15, the samples grown in the summer–autumn growing season with a 15 cm diameter head size. S11, the samples grown in the summer–autumn growing season with an 11 cm diameter head size. Student’s *t*-test is used to compare the data of two groups. Significant difference at *p* < 0.01 or *p* < 0.05. No statistically significant difference (n.s.). Number of replicates *n* = 8–10.

**Table 2:** The content of individual phenolic compound in broccoli head samples from different growth seasons and head sizes.

Samples	Individual Phenolic Content (μg/g FW)					
	Ferulic Acid	Catechin	Quercetin	Caffeic Acid	Chlorogenic Acid	Rutin
W15	19.53 ± 1.28	50.64 ± 10.93	21.56 ± 6.83	6.70 ± 1.25	150.29 ± 7.21	117.27 ± 3.82
S15	23.20 ± 0.77	54.68 ± 2.43	24.89 ± 4.72	9.45 ± 2.02	152.03 ± 9.04	83.85 ± 11.71
S11	34.01 ± 0.36	71.76 ± 15.41	44.60 ± 15.37	8.02 ± 1.29	130.66 ± 2.69	107.89 ± 2.04
W15 vs. S15	*p* < 0.05	n.s.	n.s.	n.s.	n.s.	*p* < 0.01
S15 vs. S11	*p* < 0.01	n.s.	n.s.	n.s.	*p* < 0.05	*p* < 0.05

Notes: Data are expressed as mean ± SEM. W15, the samples grown in the winter–spring growing season with a 15 cm diameter head size. S15, the samples grown in the summer–autumn growing season with a 15 cm diameter head size. S11, the samples grown in the summer–autumn growing season with an 11 cm diameter head size. Student’s *t*-test is used to compare the data of two groups. Significant difference at *p* < 0.01 or *p* < 0.05. No statistically significant difference (n.s.). Number of replicates *n* = 3.

## Data Availability

The data presented in this study are available upon request from the corresponding author.

## Supplementary Materials

The following supporting information can be downloaded at https://www.mdpi.com/article/10.3390/plants13101329/s1, Table S1 Temperature of two growing seasons in Xiangshui county (119°29′ E, 33°56′ N, Jiangsu, China). Table S2: Linear regression data for ferulic acid, catechin, quercetin, caffeic acid, chlorogenic acid, rutin, and glucoraphanin in broccoli head samples; Table S3: Condition parameters of mass spectrum analysis; Table S4: Comparison of mineral content of broccoli heads with different growing seasons and organ sizes; Table S5: The principal component analysis (PCA) of individual phenolic compounds and the calculation of comprehensive scores in the three groups; Table S6: The PCA of six phytosterol contents and the calculation of comprehensive scores in the three groups (W15, S15, and S11); Table S7: The PCA of the mineral contents and calculation of comprehensive scores in the three groups (W15, S15, and S11).
