# Supplementary material for: Profiling the Bioactive Compounds in Broccoli Heads with Varying Organ Sizes and Growing Seasons
Source: Plants (Basel). 2024 May 11;13(10):1329. doi: 10.3390/plants13101329 (PMC11125000; doi:10.3390/plants13101329)
Supplement: Supplementary file 1 [file plants-13-01329-s001.zip › plants-2979051-supplementary.pdf]

Supplemental Table S1 Temperature of two growing seasons in Xiangshui county (119°29′ E, 33°56′ N, Jiangsu, China)

Growing season	Month	Average high temperature	Average low temperature	Extreme heat	Extreme low temperature
Winter-spring growing season	January	7℃	-3℃	16℃	-12℃
	February	11℃	1℃	17℃	-4℃
	March	13℃	4℃	22℃	-2℃
	April	19℃	9℃	24℃	3℃
Summer-autumn growing season	July	31℃	23℃	35℃	21℃
	August	29℃	23℃	32℃	20℃
	September	27℃	31℃	31℃	16℃
	October	21℃	13℃	32℃	6℃

Note: temperature data from the website (https://www.tianqi24.com/xiangshui/history202107.html).

Supplemental Table S2 Linear regression data for ferulic acid, catechin, quercetin, caffeic acid, chlorogenic acid, rutin, and glucoraphanin in broccoli head samples.

**Compounds**	**Regression equation**	R^2
Ferulic acid	Y=8.2453x-25.526	0.9998
Catechin	Y=1.7503x-8.2948	0.9999
Quercetin	Y=3.9828x-10.581	0.9999
Caffeic acid	Y=9.207x-5.8537	0.9994
Chlorogenic acid	Y=4.952x-64.614	0.9981
Rutin	Y=2.1049x+16.626	0.9977
Glucoraphanin	Y=3.5773x-11.947	0.9999

Supplemental Table S3 Condition parameters of mass spectrum analysis

Compound	Retention time （min）	Parent ion (m/z)	Daughter ion (m/z)	Fragmentor voltage（v）	Collision energy（eV）
Ergosterol	13.827	379.4	125.0	125	10
			69.0	125	25
Brassicasterol	17.421	381.4	147.0	115	25
			69.0	115	35
Cholesterol	17.785	369.3	161.3	100	32
			147.2	100	33
Campasterol	21.978	383.4	161.1	130	20
			95.1	130	40
Lanosterin	22.044	409.4	149.0	120	25
			109.0	120	30
Stigmasterol	22.817	395.5	147.3	100	25
			83.1	100	20
Beta-sitosterol	27.347	397.5	161.0	100	25
			135.1	100	25
Beta-sitosteryl acetate	32.678	399.5	137.3	110	25
			95.3	110	25

Supplemental Table S4 Comparison of mineral content of broccoli heads with different growing seasons and organ sizes.

	W15	S15	S11
**phosphorus** (μg/g)	8761±359.43	9529.40±252.74	10207.80±705.70
**zinc** (μg/g)	8.50±2.17	8.20±2.28	5.00±0.71
**potassium** (μg/g)	2284.50±52.92	2261.00±87.39	2230.20±162.93
**sodium** (μg/g)	76.50±6.77	74.00±10.51	68.00±5.15
**magnesium** (μg/g)	155.67±11.96	166.00±6.40	167.20±6.87
**calcium** (μg/g)	326.00±75.48	256.80±12.32	286.80±10.33
**iron** (μg/g)	119.50±19.55	326.80±129.88	120.40±5.03

Notes: the mean ± SEM are shown. Number of replicates n = 6-8.

Supplemental Table S5 The principal component analysis (PCA) of individual phenolic compound and calculation of comprehensive scores in the three groups.

Samples	Comprehensive average scores
**S11**	0.0433
**S15**	0.0067
**W15**	-0.0500

Supplemental Table S6 The PCA of 6 phytosterol contents and calculation of comprehensive scores in the three groups (W15, S15, and S11).

Samples	Comprehensive average scores
**S11**	0.0475
**S15**	0.0410
**W15**	-0.0780

Supplemental Table S7 The PCA of the mineral contents and calculation of comprehensive scores in the three groups (W15, S15, and S11).

Samples	Comprehensive average scores
**S15**	0.0220
**W15**	0.0083
**S11**	0.0340
